# Targeted histological evaluation shows high incidence of actinomyces infection in medication-related osteonecrosis of the jaws

**DOI:** 10.1038/s41598-022-07375-1

**Published:** 2022-03-01

**Authors:** Andrea Brody, Bálint Scheich, Csaba Dobo-Nagy

**Affiliations:** 1 Department of Oral Diagnostics, Faculty of Dentistry, Semmelweis University, Szentkirályi u. 47, Budapest, 1088 Hungary; 2 Department of Pathology and Experimental Cancer Research, Faculty of Medicine, Semmelweis University, Üllői út 26, Budapest, 1085 Hungary

**Keywords:** Diseases, Medical research, Pathogenesis

## Abstract

Medication-Related Osteonecrosis of the Jaws (MRONJ) is a difficult-to-treat complication of the therapy of osteoporosis and some malignancies cured with bisphosphonates and antiresorptive drugs. The pathomechanism is unclear, but there is increasing observation that Actinomyces infection may play a role in its development and progression. The aim of our study was to demonstrate that histological examination using a validated triple staining procedure for Actinomyces bacteria strains can detect a high rate of Actinomyces infection in patient's samples with MRONJ. 112 previously hematoxylin-eosin (HE) stained samples submitted with the clinical diagnosis of MRONJ were re-evaluated histologically using an appropriate triple special staining validated for the identification of Actinomyces infection. During the first evaluation, when pathologists did not specifically look for Actinomyces, only 8.93% of the samples were reported as positive. In contrast, re-evaluation with triple staining provided a yield of 93.7% positive samples, therefore, we suggest the triple special staining to be standard in MRONJ histology evaluation. These results show that if the clinician suspects Actinomyces infection and brings this to the attention of the pathologist, it could significantly increase the number of correct diagnoses. It serves as an aid for clinicians in therapeutic success of MRONJ by selecting a long-term adequate antibiotic medication which is suitable for the elimination of actinomyces infection.

## Introduction

Medication-Related Osteonecrosis of the Jaws (MRONJ) was first described in 2003 as a complication associated with the treatment of osteoporosis and malignant diseases of the bone^1–4^. MRONJ is a condition characterized by a bone lesion that does not heal for at least 8 weeks, the patient has previously received or is receiving antiresorptive (bisphosphonate) agents or angiogenesis-inhibitor, and has no history of malignancy or radiation of the jawbones^5^.

Although it is well established that MRONJ is a complication of the above-mentioned therapies, the exact pathogenesis is unknown and seems to be multifactorial^6^. The altered remodelling and reduced blood supply impair the immune defense of the bone^7–10^. This mechanism is suggested to facilitate the development of osteomyelitis as a result of mechanical trauma and infection^11–14^, which may progress to osteonecrosis due to the poor regenerative potential caused by these factors^15^.

A distinction is made between low and high-risk groups for MRONJ. The severity of risk depends on how long the antiresorptive treatment has been applied, the route and dose of administration—the risk of developing MRONJ is 100–1000 times higher after intravenous administration^5,16–18^, the severity of the underlying disease, the patient's immune status, the comorbidities (renal failure, diabetes) and the concomitant use of other therapies (steroids, estrogen receptor inhibitors, radiotherapy). According to a retrospective analysis, about 0.5% of the Hungarian population receives bisphosphonate treatment for some reason. The incidence of MRONJ was found to be 0.9% in patients taking bisphosphonates for oncological indications and 0.1% in patients with non-malignant diseases^19^.

There is consent that one of the key factors in the development of MRONJ is bacterial infection, the source of which is the oral flora, including Actinomyces strains^8,9,20^ which are present as normal inhabitants in the oral cavity. In cases where it was detected, the average treatment time for MRONJ was longer^21,22^.

Actinomyces species are microaerophilic or anaerobic, opportunistic, Gram-positive non-spore-forming bacteria. They form radially arranged branched filamentous colonies—hence the name ‘ray fungus’ given to them upon discovery—and play a key role in biofilm formation and thus in the formation of dental plaque^12,23–25^. They are characterized by low virulence and lack of hyaluronidase enzymes, so they do not penetrate the intact mucosa. Disease development requires a damage to the integrity of the mucosa, as well as the presence of co-pathogenic bacteria (Streptococcus, Fusobacterium, Porphyromonas, etc.), creating an anaerobic environment suitable for Actinomyces strains^26,27^. The most relevant pathogenic species are Actinomyces israelii, Actinomyces viscosus, Actinomyces meyeri, Actinomyces naeslundii and Actinomyces gerencseriae^28,29^.

There are many similarities in the clinical features and morphology of jaw necrosis related to various etiologies including MRONJ, osteoradionecrosis, osteomyelitis, cemento-osseous dysplasia (COD) induced osteomyelitis and chronic diffuse sclerotizing osteomyelitis^11,27,30–35^. The majority of these cases develop following an injury disrupting mucosal integrity, and Actinomyces colonies are usually found in the necrotic area of the bone. Poor healing propensity, and a high relapse rate are also characteristic. However, it is unclear whether Actinomyces strains are causal factors in the development of MRONJ, osteomyelitis, osteoradionecrosis and COD associated bone necrosis, or colonize the necrotic area later causing further damage to surrounding tissues^36,37^.

The reported proportions of MRONJ samples with a histological confirmation of the presence of Actinomyces is markedly variable in the literature, ranging from almost 100% to around 12%^13,21,38^. However, many of them did not mention Actinomyces among the detected bacterial strains at all. It is assumable that the results were highly dependent on whether Actinomyces were specifically searched for using appropriate special stains, or they were recognized as a random finding. When choosing the test method, it is important to consider that microbiological culture is limited in its ability to detect Actinomyces strains and often gives false negative results^8,15,21,29,32,39^. This may explain why some authors have found a much lower rate of Actinomyces infection in MRONJ^25^.

## Objectives

We aimed to re-evaluate hematoxylin–eosin (HE) stained histological samples from patients diagnosed with MRONJ for the presence of Actinomyces.

## Materials and methods

### Study design and clinical data

Samples were obtained from patients diagnosed with MRONJ between 2011 and 2020 at the Department of Oro-Maxillofacial Surgery and Stomatology and Oral Diagnostics Department, Faculty of Dentistry, Semmelweis University, without any selection. The involved patients belonged to stage 2 and 3 according to American Association of Oral and Maxillofacial Surgeons (AAOMS) classification^5^.

In accordance with the recent guidelines, surgical removal of sequesters was completed with antibacterial treatment as follows: patients started antibiotic therapy 3 days before surgery and continued for 10 days in the postoperative period. In cases when appropriate microbiological data were available, definitive antibiotic therapy was used based on the sensitivity. In other cases, the empirical therapy included amoxicillin-clavulanic acid (875/125 mg, 2 × 1) as the first choice, or clindamycin (300 mg 4 × 1) or doxycycline (100 mg, 2 × 1) in patients with penicillin allergy. Cooling of the area and the use of 0.2% chlorhexidine mouth rinse at least 3 times a day for one week after surgery was recommended^40^.

Altogether 117 samples of 83 patients from the archives of the Department of Pathology and Experimental Cancer Research, Semmelweis University from 2011 to 2020 were re-evaluated. The study design was approved by the local Ethics Committee (SE RKEB 137/2020). The data of all patients were anonymized and procedures were in line with the requirements of the Helsinki Declaration. Due to the retrospective study design there was no need for ethical approval of any informed consent. Hereafter we use the term “evaluation” for the original hematoxylin-eosin (HE) study and “re-evaluation” for the second examination with specific stains for Actinomyces. A total of 117 archived samples were compared with the original histological findings during the re-evaluation. 3 samples were excluded due to the lack of unambiguous osteonecrosis and 2 other due to the insufficient bone content for further analysis. Finally, 112 archived samples were involved in the study.

All of the involved patients were treated with bisphosphonates, 101 cases with oncological indication, and 11 cases with osteoporosis. The latter group received bisphosphonate therapy only. Multiple (2–4) specimens were available from 33 patients due to persistent or recurrent lesions.

We compared the histological data with available microbiological results of the patients included in this study. Microbiological examination was performed in 39 cases within 3 months before or after the operation. For the calculation of sensitivity and specificity we made a 2 × 2 table with groups of subjects divided according to the triple staining as reference method in columns, and categories according to microbiological results in rows.

### Histological analysis

All the included samples were obtained from the surgical treatment of osteonecrosis following bisphosphonate treatment and the clinical diagnosis was MRONJ. Sections from the decalcified, formalin-fixed paraffin-embedded biopsy were stained with HE. Histological criteria included the presence of completely necrotic bone trabeculae and bacterial aggregates along with variable inflammatory infiltrates as it was previously described. During the re-evaluation, beside the HE histomorphology, the presence of Actinomyces was confirmed using Gram (Bio-Optica, Milano, Italy), periodic acid—Schiff (PAS; Surgipath Schiff reagent, Leica Biosystems, Richmond, USA), and Grocott’s methenamine silver (GMS; Biognost, Zagreb, Croatia) staining. Slides were evaluated using a Nicone Eclipse E600 POL microscope first, then the most representative samples were digitalized using a Pannoramic digital slide scanner (3DHISTECH Ltd., Hungary). Image acquisition was performed using the Case Viewer software (3DHISTECH Ltd., Hungary).

Samples containing the characteristic filamentous bacterial colonies (“sulphur granules”) showing positivity with all three stainings were regarded as „positive” to Actinomyces.

Original routine histological evaluation had been performed by 14 different pathologists in the described time period and the clinical diagnosis of osteonecrosis was confirmed in all cases. Re-evaluation of the samples was performed by 1 trained pathologist focusing on the presence of Actinomyces infection. During the re-evaluation, the examiner was blinded regarding the previous pathology report of presence or absence of Actinomyces or any microbiological data. To test the examiners reliability, he performed a repeated re-evaluation of the same samples, one and a half year following the first re-evaluation (the examiner was masked to the previous results). The intraobserver agreement was calculated with Cohen’s kappa; value is 0.93 (95% truncated CI 0.79–1). That indicates good agreement.

In the samples containing both viable and non-viable bone tissues the two areas were also compared from the perspective of Actinomyces content. Bone was considered vital when viable osteocytes were visible in the lacunae.

### Statistical analysis

SPSS Statistics 27 (IBM corp., USA) was used for statistical analysis. Group comparisons were performed using the Related-Samples McNemar Change Test with 95% confidence interval.

The intraobserver agreement was assessed by using Cohen’s kappa with its 95% confidence interval was determined; we included the data to a useable structure to *dncs_data1.xlsx*, that was used as input data. The confidence interval limits was truncated to 0 or 1 if the limits are below 0 or over 1. The calculations were made in *R* (R Core Team 2021, v4.1.1) using the psych (Revelle 2021.2.1.9) package.

### Ethical approval

The study design was approved by the *Semmelweis University Regional and Institutional Comitte of Science and Research Ethics 137/2020* (Hungary). Due to the retrospective study design there was no need for ethical approval of any informed consent: *Semmelweis University Regional and Institutional Comitte of Science and Research Ethics 155/2012* (Hungary). The study was done according to the declaration of Helsinki.

## Results

**Figure 1:**
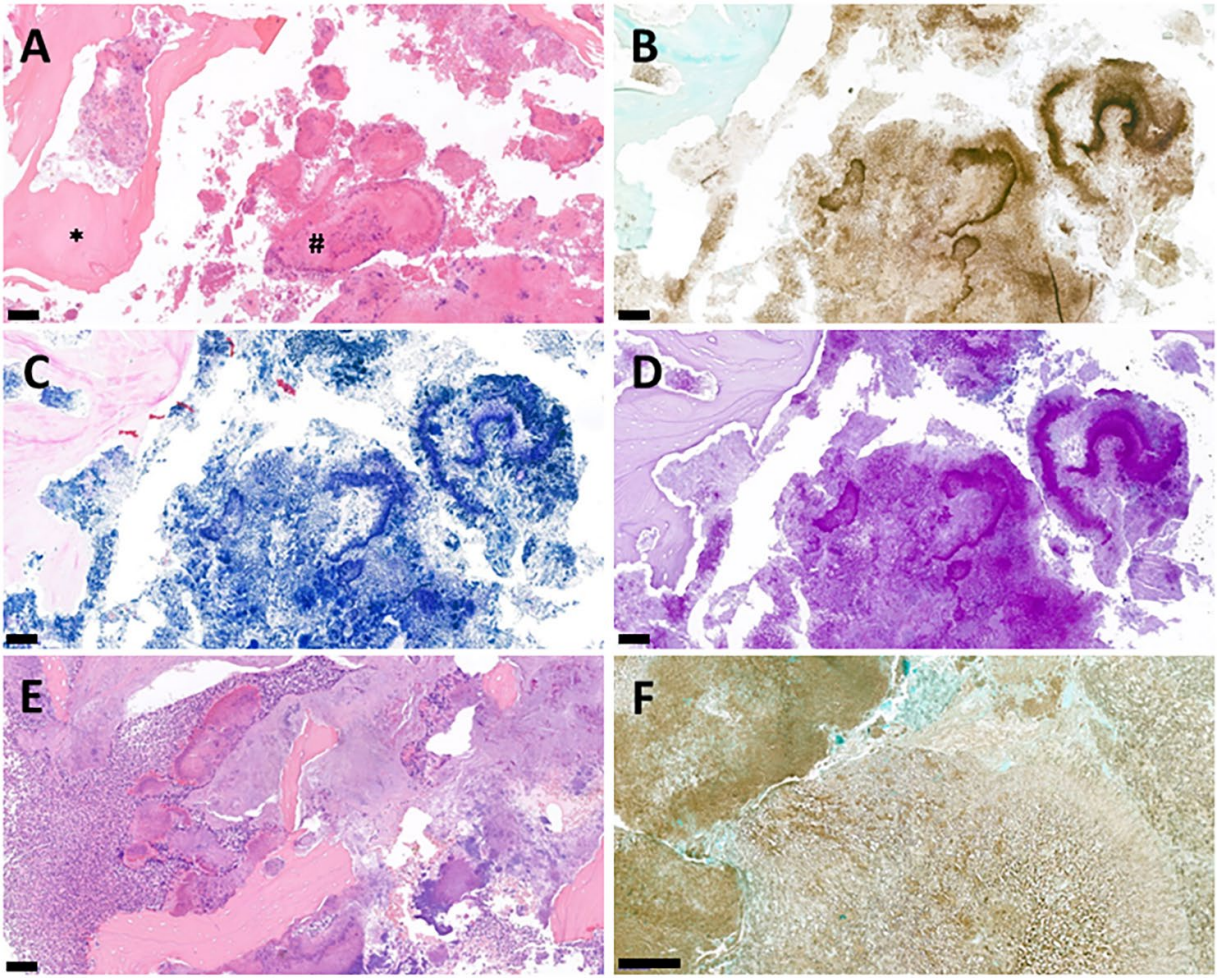
Histological characteristics of the MRONJ samples. (**A**) Histological appearance of a “positive” sample with necrotic bone trabeculae (*) and characteristic Actinomyces aggregates (#) on HE-stained section. (**B**) GMS- (**C**) Gram- and (**D**) PAS-stained sections from the same specimen showing the radial arrangement of the bacterial filaments. (**E**) Representative HE-stained section from a case with pronounced inflammatory infiltrate (left side) also containing rather uninvolved necrotic bone (right side). (**F**) A GMS-stained section of a specimen containing fungal hyphae as well. (Scale bar: **A**, **B**, **C**, **D** and **E** 100 μm; **F** 50 μm).

**Figure 2:**
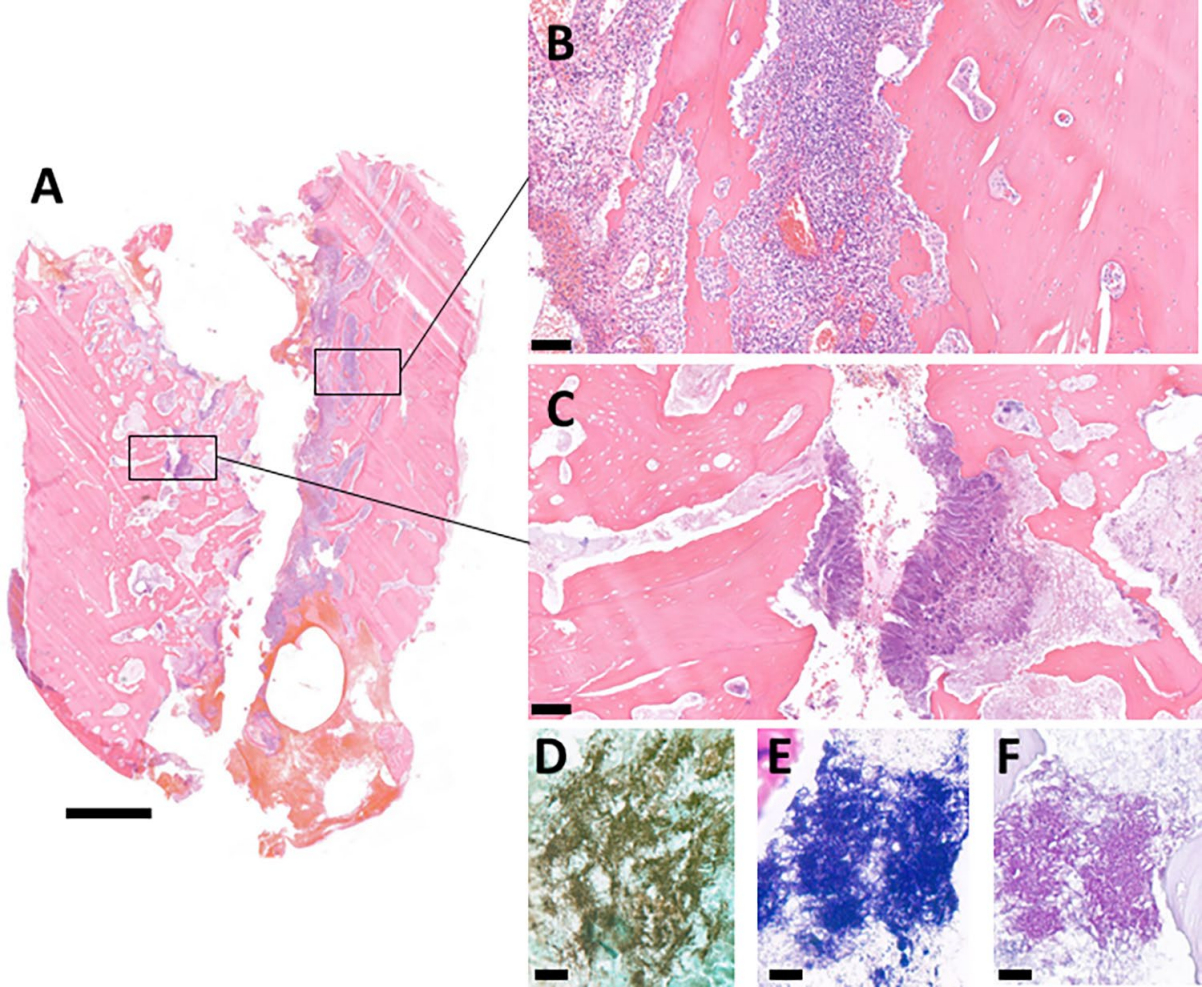
(**A**) Full cross section of a mandible resection specimen containing both (**B**) viable bone only with signs of inflammation and (**C**) necrotic bone containing Actinomyces colonies. (**D**) GMS- (**E**) Gram- and (**F**) PAS-stained representative parts. (Scale bar: **A** 2 mm; **B**,**C** 100 μm; **D**–**F** 20 μm).

The evaluated samples contained necrotic, acellular bone trabeculae with bacterial aggregates between them (Fig. 1.), usually surrounded by only mild or even absent inflammatory infiltrate (Fig. 1A). Reactive changes of the bone showed similar characteristics and were absent in the majority of cases. Actinomyces aggregates showed the characteristic radial arrangement on HE stained sections in “positive” samples (Fig. 1A), along with PAS, GMS and Gram positivity (Fig. 1C–E, respectively). In cases showing more severe inflammatory reaction, the infiltrate was usually confined to certain areas of the specimen and uninvolved necrotic bone was also present (Fig. 1E). It could also be demonstrated in some specimens that Actinomyces was present in the necrotic bone, while in viable and inflamed areas, the bacterial colonies were not detected (an example is demonstrated on Fig. 2). In 3 cases, fungi were also detectable (Fig. 1F).

Among the 112 archived samples 102 (91.07%) were reported as Actinomyces negative during the first routine histological evaluation with HE stainings, of which 95 were found to be positive during the re-evaluation with triple specific stains. Compared with the original report 7 samples (6.25%) were found to be negative during the re-evaluation. In the evaluation, the presence of Actinomyces was detected in 10 samples (8.93%) and this result was confirmed in all cases in the re-evaluation, so in the end a total of 105 (93.75%) samples were found to be positive. The result proved highly significant (p = 0.0000) (Table 1).

**Table 1:** Original evaluation was performed on HE-stained sections, while the re-evaluation was specifically focused on the presence of Actinomyces and complements with special stains.

Proportion of Actinomyces positive and negative samples				

112 samples	Negative	%	Positive	%
Evaluation	102	91,07	10	8,93
Re-evaluation	7	6,25	105	93,75
p-value	0,0000			

Actinomyces colonies could be detected in 105 (93.75%) of the re-evaluated cases, while it was described only in 10 (8.93%) samples in the original evaluation.

As a result of the microbiological examination the presence of Actinomyces could be detected in cultures in only 2 of 39 cases (5.13%), all of which were evaluated histologically as “positive” during the re-evaluation. No samples were histologically negative and microbiologically positive. 2 of 39 samples were negative with both histological and microbiological examinations. Comparison of triple staining histology and microbiology results resulted in high specificity (1) and very low sensitivity (0.054). Negative predictive value of routine microbiological test was 0.054.

Beside the two Actinomyces positive culture we found Fusobacteria, Prevotella, Eikenella, and Enterobacteria strains. In the remaining 35 histologically positive cases, the following bacteria were found in the microbiological culture Prevotella (12), Fusobacteria (5), Parvimonas (5), Veillonella (4), Eikenella (2), Streptococcus (7) Klebsiella (6) Staphylococcus (4), Enterobacter (2), Enterococcus (1) Haemophylus (2) and Citrobacter (1) strains.

## Discussion

MRONJ is a multifactorial, not a life-threatening disease, but significantly impairing the quality of life. There are still many questions about the pathogenesis, but most authors agree that infections may play a major role in its development. Many of them have raised the possibility that Actinomyces strains are potentially involved in this process, in addition to other bacteria^4,9,13,20,41–43^. A highly variable proportion of Actinomyces positivity can be found in the literature, however, in the studies with lower prevalence the methods of Actinomyces detection, including microbiology or HE staining of histological samples, were probably inadequate regarding sensitivity^25^. Cerrato's analysis of 30 publications showed that Actinomyces positivity occurred in 96.4% to 25.4% of the samples^7^. These variances are presumably related not only to methodological differences but whether the study design was focused on the presence of Actinomyces or not.

In our study, we re-evaluated the histopathologycal samples in our database that had been received with a MRONJ diagnosis over a 10-year period to examine the frequency of Actinomyces. To the best of our knowledge there has been no study to date that has re-stained and re-evaluated previously tested samples. The re-evaluation of archived samples with a special focus on and using appropriate stains for Actinomyces, showed positivity in 93.75% compared to the 8.93% of the original routine evaluation. There were no cases where the sample initially found to be positive was found as negative in the re-evaluation. Only 7 out of the 112 samples proved to be negative in both the routine and the re-evaluation.

These results suggest that one of the reasons for the significant discrepancy in the literature reports on the prevalence of Actinomyces may be a methodological problem. The routinely used HE staining resulted in a sparse detection of Actinomyces, which therefore seems more likely to be an accidental finding on MRONJ samples.

The sensitivity of microbiological cultures is very poor regarding the detection of Actinomyces species^27,29,51,52^. Microbiological results were available in case of 39 samples in the patient’s records. Actinomyces could only be detected in two samples, representing a prevalence of 5.13% versus the 93.75% result of histology. All two samples were evaluated histologically as “positive” during the re-evaluation. These data confirm the well-known fact that microbiology is not a proper method to detect Actinomyces and underlie the importance of adequate histological assessment.

In dental plaques, Actinomyces species create co-aggregates with other bacteria, mainly Fusobacteria, Prevotella, Eikenella and Veillonella strains, during the formation of the biofilm. The role of this cooperation seems to be basically important in the pathogenesis of osteomyelitis as well, in which these species adhere to collagen fibers and promote the development of osteonecrosis^43,44^. During the review of the microbiological data, we found that Prevotella, Fusobacteria, Parvimonas, Veillonella, Eikenella, Staphylococcus, Streptococcus and Klebsiella species were most frequently found in the samples.

The potential causal role of Actinomyces in the pathogenesis of MRONJ is still an unresolved issue^9,15,36,37^, however, and increasing number of authors suggest a causal role of Actinomycetes in the development of MRONJ based on the higher prevalence detected by histological evaluation^7,20,22,37,39,41,44^. Russmueller et al. found a high (89%) prevalence and concluded that Actinomyces strains play a prominent role in MRONJ and will change our understanding of it. In a systematic review published in 2020, Cerrato concluded that osteomyelitis caused by Actinomyces and MRONJ may have a common origin with the notion that the lower prevalence reported in earlier series is at least partially related to methodological problems. Our study supports Cerrato's latter view.

The therapy of MRONJ is generally based on the recommendation of AAOMS (American Association of Oral and Maxillofacial Surgeons). According to the AAOMS principles, systemic antibacterial therapy is recommended from stage 3, but the above mentioned position paper does not mention the length of antibiotic therapy.

The MRONJ protocols of different countries usually recommend a few weeks of antibiotic treatment after surgery^12^. In contrast, the recommended antibiotic treatment protocols for actinomycosis of the jawbone start with intravenous broad-spectrum antibiotics (amoxicillin-clavulanic acid, ampicillin-sulbactam) for 3–4 weeks and continue with oral penicillin for 9–12 months or longer^45,47,48^, whereas Actinomyces strains are sensitive to beta-lactam compounds, but these compounds show very poor potential to penetrate fibrotic, necrotic and inflamed tissues and large bacterial aggregates^49^. Therefore, following a few weeks of intravenous therapy penicillin V can be used for the long term, significantly decreasing the incidence of gastrointestinal side effects compared to penicillins with beta-lactamase inhibitors^27,43,44,47,50–52^.

The proportion of Actinomyces positive samples in our study were undoubtedly higher in comparison with some previous reports, although not far from the result of *Rusmueller *et al*.,* or even lower than others, Anavi-Lev, Lee, Naik, Hansen, Franco-Pretto^16,37,39,45,46,53^. However, the antibiotic regime used in our patients is unlikely to affect the proportion of Actinomyces positive cases, since it is started only three days before the sampling. It is important to note that these short preoperative treatment is insufficient to eliminate Actinomyces.

Summarized, it would be important for the clinician to consider the possibility of Actinomyces infection in all MRONJ samples and bring this to the attention of the pathologist, who will then search for it using validated methods. This will presumably increase the frequency of detected Actinomyces infections and allow for the selection of an appropriate therapy. The focused histological examination, including triple stains and appropriate clinical issues, can be significant, and result in much higher detection rate of Actinomyces in comparison with isolated HE staining. This is highlighted by the fact that the microbiological detection of Actinomyces using conventional techniques shows poor sensitivity.

Additionally, long-term antibiotic treatment protocols should be considered in the postoperative pharmacotherapy of MRONJ, in order to eradicate Actinomyces species. The currently applied too short antibiotic therapy may contribute to the large number of therapeutic failures.

## Data Availability

The datasets generated and analysed during the current study are available from the corresponding author on reasonable request.
